# A new species of *Euscorpius* Thorell, 1876 (Scorpiones, Euscorpiidae) from Turkey

**DOI:** 10.3897/zookeys.219.3597

**Published:** 2012-09-04

**Authors:** Gioele Tropea, Ersen Aydın Yağmur, Halil Koç, Fatih Yeşilyurt, Andrea Rossi

**Affiliations:** 1 Società Romana di Scienze Naturali, Rome, Italy; 2 Alaşehir Vocational School, Celal Bayar University, Manisa, Turkey; 3 Sinop University, Science and Art Faculty, Biology Department, Sinop, Turkey; 4 Kırıkkale University, Science and Art Faculty, Biology Department, Zoology Section, Kırıkkale, Turkey; 5 Aracnofilia, Centro Studi sugli Aracnidi, Massa, Italy

**Keywords:** Scorpion, *Euscorpius*, new species, Turkey

## Abstract

A new species of the genus *Euscorpius* Thorell, 1876is described based on specimens collected from Dilek Peninsula (Davutlar, Aydın) in Turkey. It is characterized by an oligotrichous trichobothrial pattern (*Pv*= 7, *et*= 5/6, *eb*= 4) and small size. *Euscorpius (Euscorpius) avcii* **sp. n.** is the first named species of the subgenus *Euscorpius* from Turkey.

## Introduction

The genus *Euscorpius* Thorell, 1876 is one of the most studied taxa of scorpions. According to the present taxonomy, it includes 18 species grouped in four subgenera (*Alpiscorpius* Gantenbein et al. 1999; *Euscorpius* Thorell, 1876; *Polytrichobothrius* Birula, 1917; *Tetratrichobothrius* Birula, 1917) (Fet et al. 2004; Vignoli and Salomone 2008; Tropea 2012). However, its taxonomy is still not totally clear, especially in the Balkans and Turkey but also in Western Europe. The *Euscorpius* populations of Turkey have been poorly studied up to now, and only two valid species are recognized; *Euscorpius (Polytrichobothrius) italicus* (Herbst, 1800) and *Euscorpius (Alpiscorpius) mingrelicus* (Kessler, 1874). The latter is a species complex with six subspecies in Turkey (*Euscorpius mingrelicus mingrelicus* (Kessler, 1874), *Euscorpius mingrelicus ciliciensis* Birula, 1898, *Euscorpius mingrelicus phrygius* Bonacina, 1980, *Euscorpius mingrelicus ollivieri* Lacroix, 1995, *Euscorpius mingrelicus legrandi* Lacroix, 1995,and *Euscorpius mingrelicus uludagensis* Lacroix, 1995) that need clarification. Presence of the *“carpathicus* complex” have been reported by several authors; Hadži (1930) reported it from İstanbul; Schenkel (1947) from Havza (Samsun); Vachon (1951) from Acıpayam and Honaz Mountain (Denizli), Eğridir (Isparta), Korikos (Mersin) and İstanbul; Tolunay (1959) from Sinop; Kinzelbach (1975, 1982) from Amasya, the Middle Taurus, Borçka (Artvin), Çanakkale, Trakya and Efes (Izmir); Karatas (2006) from Marmara area, Sinop, Ada vicinity, Alanya, Avsallar, Fethiye and Kelebekler Valley; Koç and Yağmur (2007) from Dilek peninsula.

Kinzelbach (1975) divided *Euscorpius carpathicus* into two species, *Euscorpius carpathicus* and *Euscorpius mesotrichus* Hadži, 1929. According to Kinzelbach (1975), *Euscorpius tergestinus* is a synonym of *Euscorpius mesotrichus*, but the latter name is not available because it is a junior homonym of *Euscorpius italicus mesotrichus* Hadži, 1929 (Di Caporiacco 1950; Fet 1997; Fet and Braunwalder 2000). *Euscorpius mesotrichus* was synonymized with *Euscorpius tergestinus* by Di Caporiacco (1950) and according to Fet and Braunwalder (2000) the correct name for this species should be *Euscorpius tergestinus*, but further studies (Gantenbein et al. 2001; Fet et al. 2003) reported that “*Euscorpius mesotrichus*” of Kinzelbach also refers to other species such as *Euscorpius balearicus* and *Euscorpius sicanus*, besides *Euscorpius tergestinus* and other forms waiting for clarification. “*Euscorpius mesotrichus*” was recorded in Turkey from Şile (İstanbul) and Prinkipos Island (Büyükada Island) in the Marmara Sea by Kinzelbach (1975).

Koç and Yağmur (2007) reported a population from Dilek Peninsula in Western Turkey as *Euscorpius* sp. (“*carpathicus* complex”). A Dilek specimen was also listed by Vignoli and Salomone (2008) as *Euscorpius*. cf. *tergestinus* (AMNH, Söke, Davutlar, 44 m a.s.l., 28.IV.2005, H. Koç). This population is described in this study as a new species, *Euscorpius avcii* sp. n. According to our preliminary studies on Turkish *Euscorpius* populations, more species and forms ranging from polytrichous to oligotrichous are present and of these, the latter exhibits diagnostic characters that appear intermediate between the subgenus *Euscorpius* and *Alpiscorpius*. The new species, *Euscorpius avcii* sp. n. is oligotrichous and differs from other forms of the genus *Euscorpius* enough to justify its description as the first species of the subgenus *Euscorpius* to be registered in Turkey.

## Materials and methods

A number of 79 specimens collected at Dilek Peninsula, in Turkey, were examined. Furthermore, 56 specimens from MZUF (*Euscorpius tergestinus* (C.L. Koch, 1837): 132/5856, 84/5847, 5848, 5861, 5862, 5863, 131/5838, 5839, 5840, 5841, 5842, 5843, 132/5854, 5856, 5857, 5860, 135/5699, 161/5850, 5851, 162/5864, 5865, 5866, 5867, 163/5987, 5988, 5889, 5990, 5991, 5992, 5993, 5994, 5995, 5996, 5997, 5998, 180/5852, 1417/5999, 6000, 6001, 6002, 6003, 6004, 6005, 6006, 6007, 6008, 6009, 6010, 6011, 6012, 165/6226, 73/6032, 1149/6238; *Euscorpius oglasae* Caporiacco, 1950 lectotype 122/5974, paralectotypes 123/5975) and 13 specimens of the private collection of Gioele Tropea (10 *Euscorpius tergestinus* (C.L. Koch, 1837) from Italy (Abruzzo, Latium and Umbria) and 3 *Euscorpius carpathicus* sensu stricto(Linnaeus, 1767) from Romania) were included in this study as comparison material.

Abbreviations: *V*: trichobothria on ventral pedipalp chela manus; *Pv*: trichobothria on patella ventral surface; *Pe*: trichobothria on the pedipalp patella external surface; *et*: external terminal; *est*: external sub-terminal; *em*: external medium; *esb*: external suprabasal; *eba*: external basal *a*; *eb*: external basal; DPS: dorsal patellar spur; DD: distal denticle; MD: median dentition; OD: outer dentition; ID: inner dentition; IAD: inner accessory denticles; AMNH: American Museum of Natural History, New York, USA; MZUF: Museo Zoologico ‘La Specola’ dell’Università di Firenze, Florence, Italy; GTC: private collection of Gioele Tropea; MTAS: Museum of the Turkish Arachnological Society; ZMSU: Zoology Museum of Sinop University; KUAM: Arachnological Museum of Kırıkkale University; ARC: private collection of Andrea Rossi.

The trichobothrial notations follow Vachon (1974). The morphological measurements are given in millimeters (mm) following Stahnke (1970). The morphological nomenclature follows Stahnke (1970), Hjelle (1990) and Sissom (1990); the chela carinae and denticle configuration follows Soleglad and Sissom (2001) and sternum terminology follows Soleglad and Fet (2003); description of hemispermatophore and terminology follows Soleglad and Sissom (2001) and Fet and Soleglad (2002).

## Taxonomy

### Family Euscorpiidae Laurie, 1896

Genus *Euscorpius* Thorell, 1876

Subgenus *Euscorpius* Thorell, 1876

#### Euscorpius avcii

Tropea, Yağmur, Koç, Yeşilyurt & Rossi sp. n.

urn:lsid:zoobank.org:act:B6799900-DF03-488A-B675-F0195AEB9825

http://species-id.net/wiki/Euscorpius_avcii

##### Type material.

**Holotype:** 1 ♂, Dilek Peninsula National Park, Canyon, Dilek Peninsula, near Davutlar Town, Kuşadası, Aydın, Turkey, 07.10.2005, leg. H. Koç (MTAS).

**Paratypes: 1.** 2 ♀♀, 3 ♂♂, Dilek Peninsula National Park, Canyon, Dilek Peninsula, near Davutlar Town, Kuşadası District, Aydın Province, Turkey, 06.11.2004, leg. H. Koç (ZMSU); 2 ♀♀, 1 sub♂, 3 sub♀, Dilek Peninsula National Park, Canyon, Dilek Peninsula, near Davutlar Town, Kuşadası District, Aydın Province, Turkey, 07.10.2005, leg. H. Koç (MZUF); same data but 1 ♂, 2 ♀ (GTC); 4 ♂♂, 3 ♀♀, Dilek Peninsula National Park, Canyon, Kuşadası District, Aydın Province, Turkey, 04.05.2011. 37°41'37"N, 27°09'37"E, 82 m, leg. E.A. Yağmur, A. Avcı and F. Yeşilyurt (MTAS); 5 ♂♂, 10 ♀♀, Dilek Peninsula National Park, Canyon, Dilek Peninsula, near Davutlar Town, Kuşadası District, Aydın Province, Turkey, 07.10.2005, leg. H. Koç (ZMSU); 3 ♀♀, Dilek Peninsula National Park, Canyon, Dilek Peninsula, near Davutlar Town, Kuşadası District, Aydın Province, Turkey, 18.06.2005, leg. H. Koç (ZMSU).

**2.** 3 ♂♂, 6 ♀♀, 5 km south of Güzelçamlı Village, Davutlar Town, Kuşadası District, Aydın Province, Turkey, 07.06.2011, 37°41'22"N, 27°13'31"E, 311 m, leg. F. Yeşilyurt and E.A. Yağmur (KUAM). Same data but 1 ♂, 1 ♀ (ARC). 3 ♂♂, 8 ♀♀. 5 km south of Güzelçamlı Village, Davutlar Town, Kuşadası District, Aydın Province, Turkey, 13.07.2010, 37°41'25"N, 27°13'53"E, 428 m, leg. F. Yeşilyurt and T. Danışman (KUAM).

**3.** 1 ♂, 8 ♀♀, Dilek Peninsula, 2 km south of Davutlar Town, pine forest, Kuşadası District, Aydın Province, Turkey, 02.07.2011, leg. E.A. Yağmur and A. Avcı (MTAS).

**4.** 6 ♂♂, 2 ♀♀, Dilek Peninsula National Park, picnic area, laurel forest, Kuşadası District, Aydın Province, Turkey, 13.08.2009, leg. E.A. Yağmur, N. Tezcan and V. Ülgezer (MTAS).

##### Etymology.

The specific epithet refers to Dr. Aziz Avcı who is a Turkish herpetologist and the new species is named after him for his kind contributions to collecting scorpion species and his friendship.

##### Diagnosis.

A small *Euscorpius* species, total length 24–28 mm. Color of adults is light brown to brown-reddish with the carapace and pedipalps darker brown-reddish, legs and telson lighter, yellowish colored. *Euscorpius avcii* sp. n. is oligotrichous; the number of trichobothria on the pedipalp manus ventral surface is 4 (3 *V + Et* 1); the number of trichobothria on the pedipalp patella ventral surface is 7 (of 78.5% of examined specimens and of 88% of pedipalps). The number of trichobothria on pedipalp patella external surface is: *eb* = 4, *eba* = 4, *esb* = 2, *em* = 4, *est* = 4, *et* = 5/6 (generally 5). The pectinal teeth count is: 7-9 (generally 8) in males, 6-7 (generally 7) in females. The telson vesicle in males is more swollen than in females, but only slightly more swollen if compared to other species of the subgenus *Euscorpius*. The pedipalps are stocky with a notch on fixed finger and scalloping of the movable finger well developed in adult males, obsolete in females. The dorsal patellar spur is weakly developed. Carinae on the metasomal segments are strongly reduced, almost smooth. Average value of the length from center median eyes to anterior margin of the carapace is equivalent to 39.20±2.0% of the carapace length. Average value of the length from center median eyes to posterior margin of the carapace is equivalent to 60.80±2.0% of the carapace length.

##### Description of the holotype male.

**Coloration:** Light brownish with carapace and pedipalps darker, brownish-reddish, legs, telson and chelicerae are lighter, yellowish-orange. Carapace slightly marbled. The coxal region is distinctly brownish-orange colored. The sternites, pectines and genital operculum are very light brownish-white (Fig. 3, 4 and 5).

**Figure 1.**
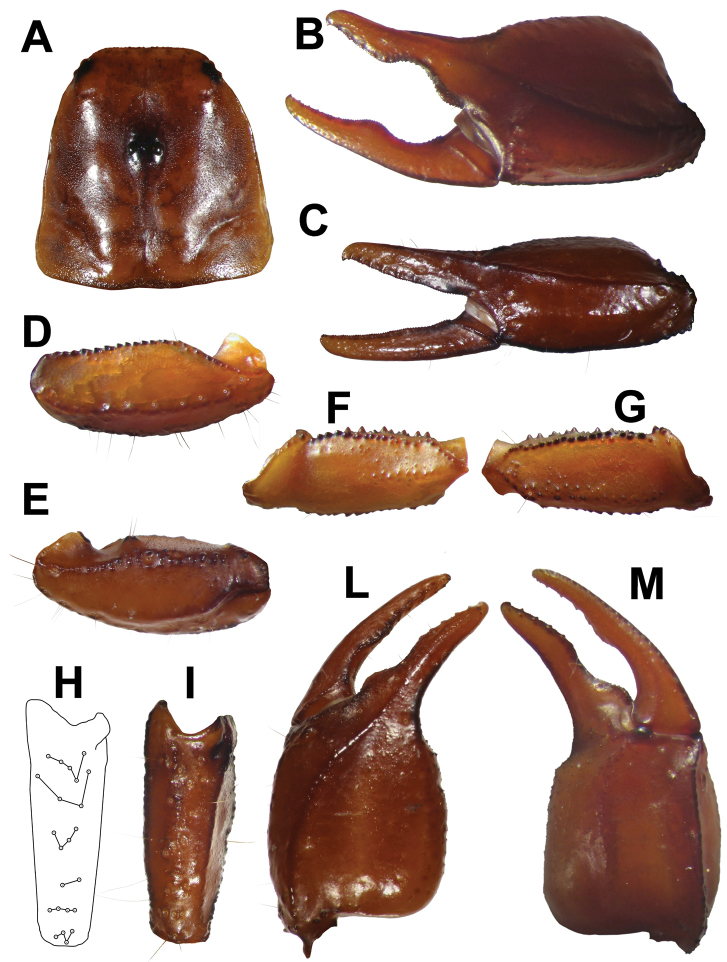
**A** carapace **B** external view of chela of the adult male **C** external view oh chela of the adult female **D** ventral view of pedipalp patella **E** dorsal view of pedipalp patella **F** ventral view of pedipalp femur **G** dorsal view of pedipalp femur **H** schematic view of trichobothrial pattern on external surface of pedipalp patella **I** view of external surface of pedipalp patella **L** dorsal view of chela **M** ventral view of chela.

**Carapace:** Length 3.70 mm; posterior width 3.75. Very slightly and finely granulated in laterally. All the furrows are shallow, only the posterior lateral furrows are slightly more marked. Distance from the center of the median eyes to the anterior margin of the carapace is equivalent to 39.62% of the prosoma; the length from the center of the median eyes to the posterior margin of the carapace is equivalent to 60.38% of the prosoma (Fig. 1A).

**Mesosoma:** Tergites veryslightly and finely granulated, almost smooth; sternites smooth. The area of overlap between the sternites is lighter in color. Pectinal teeth count is 8-9. The spiracles are very small, oval shaped and it is inclined to about 45° downwards towards outside.

**Figure 2.**
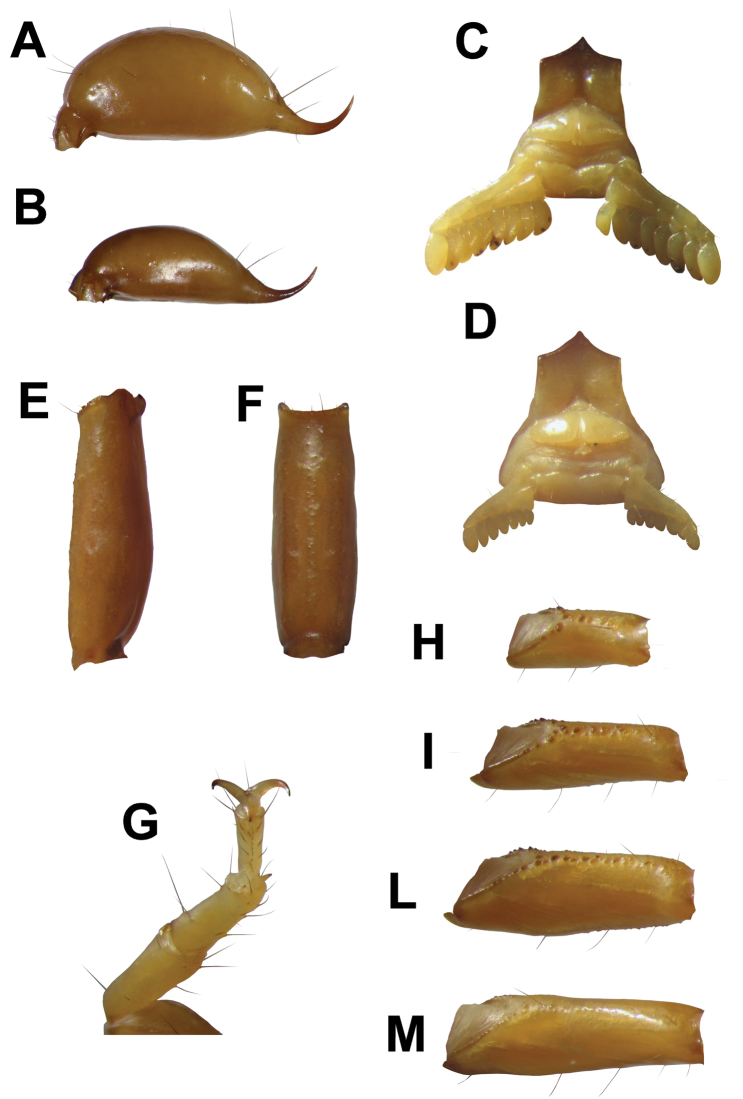
**A** telson of adult male **B** telson of adult female **C** sternopectinal area of adult male **D** sternopectinal area of adult female **E** latero-dorsal view of the metasomal segment V **F** ventral view of the metasomal segment V **G** tarsus and basitarsus **H** leg femur I **I** leg femur II **L** leg femur III **M** leg femur IV.

**Metasoma:** Medium to small size with respect to body length. Dorsal carinae from segment I–IV are almost smooth, exhibit a few distanced fine granules, obsolete or almost obsolete on the segment V; ventromedian carinae from segment I–IV absent; ventromedian carinae on segment V are formed by very fine granules. Ventrolateral carinae from segment I–IV are obsolete; on segment V they are formed by a few spaced granules (Fig. 2E,F).

**Telson:**Vesicle weakly swollen (Fig. 2A); smooth, with ventral setae of different sizes; telson height 1.37, telson length 3.65, vesicle length 2.65, vesicle width 1.40.

**Pectines:** Pectinalteeth count 8-9; middle lamellae count 5-4.

**Genital operculum:** Partially divided with genital papillae protruding; a few microsetae present.

**Sternum:** Pentagonal shape, type 2. Length similar to width, deep posterior emargination.

**Pedipalp:** Coxa and trochanter with strong granulation. Femur: dorsal internal carinae tuberculate; dorsal external carinae formed by low spaced tubercles, their size increases from distal to proximal. Intercarinal spaces bears scattered small granules, larger in the posterior proximal area. Ventral external carina is granulated in the proximal half. External median carinae serrulate, anterior median crenulate and tuberculate distally. Patella length 3.25; patella width 1.20; dorsal internal carinae crenulate. Dorsal external carinae from rough to smooth and are crenulate proximally. Ventral external carinae from smooth to rough. Ventral internal carinae serrulate. Intercarinal tegument smooth or rough. Dorsal patellar spur weakly developed (Fig. 1E).

Chelal carina *D_1_* isdistinctly strong, dark and from smooth to rough; *D_4_* is formed from scattered granules; *V_1_* isdistinctly strong, crenulate and dark; *V_3_* is formed from granules on 2/3 of length.External carina with granules on distal half. Intercarinal tegument rough or smooth except between carinae *D4* and *V3*. Movable finger dentition: MD like a straight line formed from very small denticles closely spaced and an DD on the distal tip; OD formed from 7 denticles on movable finger and 6 denticles on fixed finger, immediately outside of MD, their size increases progressively but the terminal denticle is not very pronounced; ID formed from 7 denticles on movable finger and 6 denticles on fixed finger, spaced from MD, their size increases progressively but the terminal denticle is not very pronounced; IAD on both movable and fixed finger formed from 4 small denticles.

**Trichobothria:** Chela trichobothria series *V* standard: *V* = 4-4 (3 *V+ Et*1); patella ventral (*P*v): 8-7; Patella external (*P*e): *et* = 5-5, *est* = 4-4, *em* = 4-4, *esb* = 2-2, *eba* = 4-4, *eb* = 4*-*4.

**Legs:** legs with two pedal spurs. Tarsal ventral row with 10-12 stout spinules; 3 tarsal setae flanked pairs adjacent to the ventral spinules row. Basitarsus with 6 prolateral stout spinules on leg pair I; 7 prolateral stout spinules on leg pair II; 1 prolateral stout spinules on leg pair III; absent on leg pair IV. Granulation on the leg femora II and III is more marked both dorsally and ventrally, and only ventrally on leg I. Granulation is formed from dark granules; while the granulation on the dorsal surface of the femur of leg I and on the femur of leg IV both dorsally and ventrally is weakly marked and of lighter colored granules.

**Chelicerae:** movable finger: The dorsal distal tooth is smaller than the ventral distal tooth; Ventral edge is smooth with brush-like setae on the inner part; dorsal edge has five teeth: one distal, two small subdistal, one big median and a small basal; fixed finger has four teeth: one distal, one subdistal, one median and one basal. The median and the basal are in a fork arrangement. The internal edge has brush-like setae.

**Variation:** The variation observed in 79 studied specimens (29 males, 50 females) is the follows: pectinal teeth in males: 7-7 (1/29), 8-8 (23/29), 8-9 (4/29), 9-9 (1/29); females: 6-6 (5/50), 6-7 (11/50), 7-7 (34/50); pedipalp patella trichobothria *Pv*: 8-8 (2/79), 8-7 (9/79), 7-7 (62/79), 6-7 (6/79); pedipalp patella trichobothria *Pe*: *et* = 5-5(41/79), 5-6 (19/79), 6-6 (19/79); *est* = 4-4 (79/79), *em* = 4-4 (79/79), *esb*= 2-2 (79/79), *eba* = 4-4 (79/79), eb = 4-4 (79/79). The variation in the trichobothrial pattern is within the standard values of variability and shows the stability of diagnostic characters.

##### Hemispermatophore.

Well developed lamina with well visible basal constriction, tapered distally; truncal flexure present and well developed; capsular lobe complex well developed, with acuminate process; ental channel spinose distally, exhibiting six delicate variable sized spines (Fig. 6).

**Figure 3.**
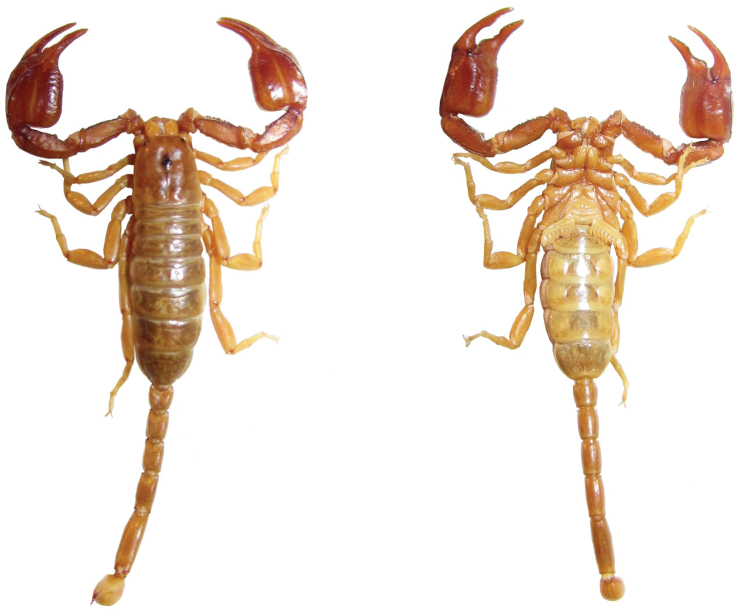
Dorsal and ventral views of *Euscorpius avcii* sp. n.male.

**Figure 4.**
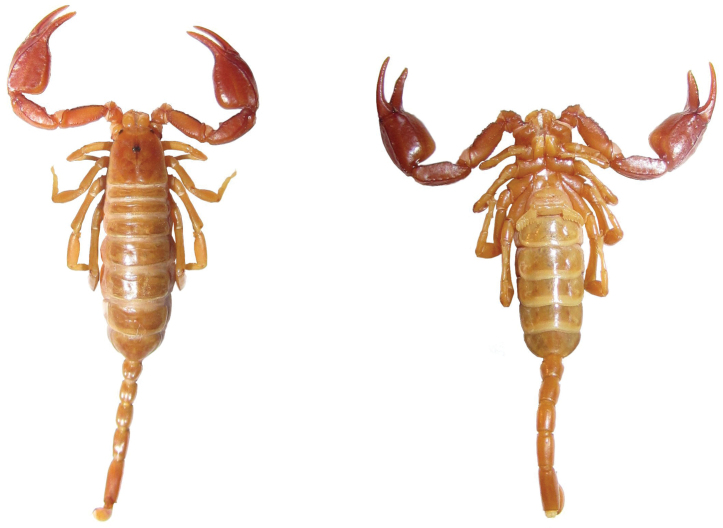
Dorsal and ventral views of *Euscorpius avcii* sp. n.female.

**Figure 5.**
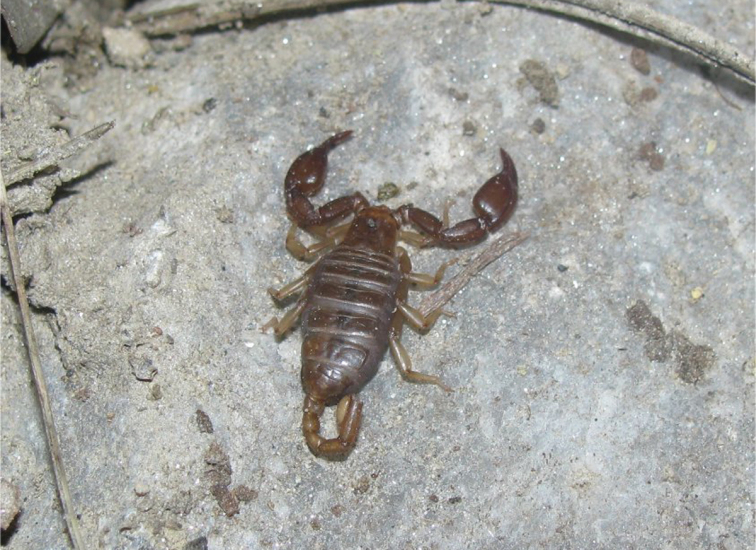
*Euscorpius avcii* sp. n. in its natural habitat.

**Figure 6.**
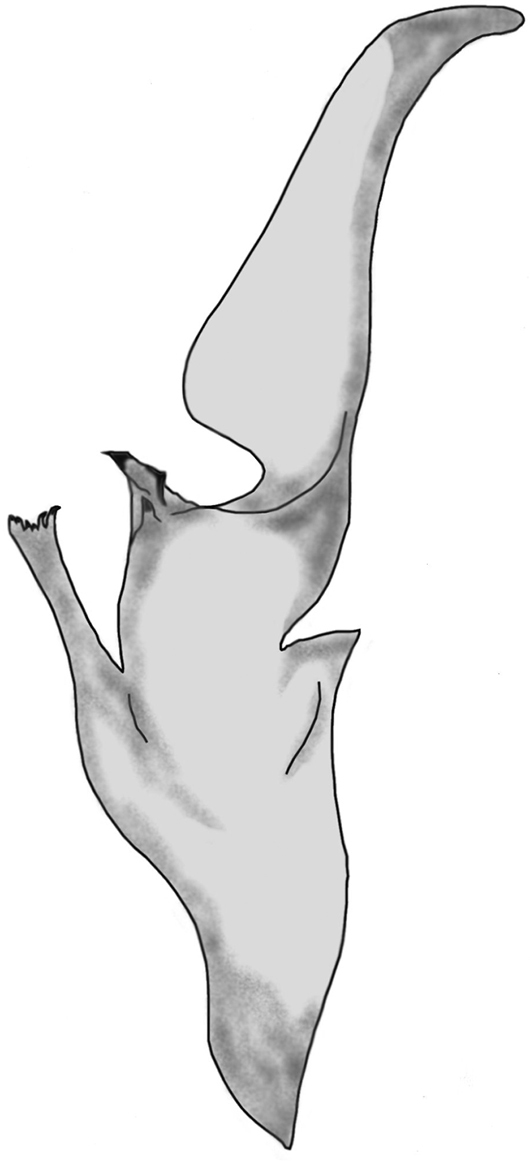
Left hemispermatophore of *Euscorpius avcii* sp. n.

## Discussion

*Euscorpius avcii* sp. n. is an oligotrichous form with *Pv* = 7 and *Pe-et* = 5/6. Most of the species belonging to the subgenus *Euscorpius* have generally higher trichobothrial numbers, with some exceptions e.g. *Euscorpius oglasae* Di Caporiacco, 1950 (*Pv* = 7, *Pe-et* = 5) (Vignoli et al. 2007) and an unnamed form from the island of Samos in Greece (*Pv* = 5, *Pe-et* = 5) (Vignoli and Salomone 2008). Kinzelbach (1975) mentions *Euscorpius carpathicus* (Linnaeus, 1767) and *Euscorpius mesotrichus* from some localities in Turkey, according to the author the specimens with *Pv* 7/8 are *Euscorpius carpathicus*, with *Pv* 10/14 are *Euscorpius mesotrichus*. According Kinzelbach (1975) *Euscorpius tergestinus* is a synonym of *Euscorpius mesotrichus*, but the latter name is not available because it is a junior homonym of *Euscorpius italicus mesotrichus* Hadži, 1929 (Di Caporiacco 1950; Fet 1997; Fet and Braunwalder 2000). *Euscorpius mesotrichus* was synonymized with *Euscorpius tergestinus* by Di Caporiacco (1950) and according to Fet and Braunwalder (2000) the correct name for this species should be *Euscorpius tergestinus*. Further studies (Gantenbein et al. 2001; Fet et al. 2003) reported that “*Euscorpius mesotricus*” of Kinzelbach also refers to other species such as *Euscorpius balearicus* and *Euscorpius sicanus* besides *Euscorpius tergestinus* and other forms that need clarification. *Euscorpius carpathicus* s. str. is now restricted to the populations of the type locality in Romania (Fet and Soleglad 2002). Among the specimens studied by Vignoli and Salomone (2008), there is one from Turkey of the AMNH collection labeled as *Euscorpius* cf. *tergestinus* (1 juvenile, Aydın Davutlar, Söke 44 m a.s.l., 28.IV.2005, H. Koç coll.) from the same population as presented in this study as a new species. The specimens of our study certainly do not fall within the range of *Euscorpius mesotrichus* “of Kinzelbach” nor in *Euscorpius carpathicus* s. str. and *Euscorpius tergestinus* s. str., as we shall see from morphological and trichobothrial data below.

*Euscorpius oglasae* has a trichobothrial pattern that is almost identical to *Euscorpius avcii* sp. n., but the morphology and geographic distribution (*Euscorpius oglasae* is endemic to the island of Montecristo in the Tyrrhenian Sea in Tuscany, Italy) make easy to separate these two species. *Euscorpius oglasae* is larger than *Euscorpius avcii* sp. n. (up to 43 mm) (Vignoli et al. 2007), the lobe of the movable finger is weak in males and obsolete in females, the chela is slender, whereas *Euscorpius avcii* sp. n. has a very pronounced lobe on movable finger and the notch on fixed finger and the chela is stocky. The DPS is more developed in *Euscorpius oglasae* as well as the granululation and metasomal carinae. *Euscorpius oglasae* has a lower pectinal teeth count, 7–7 in males and 6–6 females, whereas *Euscorpius avcii* sp. n. has 8–8 in males and 7–7 in females.

Samos is a Greek island inhabited by an unnamed oligotrichous form, similar to *Euscorpius avcii* sp. n. The Samos population is characterized by small size, stocky pedipalps and trichobothrial pattern *Pv* = 5 and *et* = 5 (Vignoli and Salomone 2008). This form therefore seems to have a lower *Pv* count and *et* constant (*Euscorpius avcii* sp. n. has *Pv* = 7 and *et* = 5/6). Samos Island is very close to the Dilek Peninsula (in some places less than two kilometers), therefore a relationship could be possible between these two populations, but because of the lack of information about the Samos form, we cannot discuss its taxonomical relationship to *Euscorpius avcii* sp. n.

*Euscorpius tergestinus* s. str. is easily distinguished from *Euscorpius avcii* sp. n., even if the color and the trichobothrial pattern *eb* = 4, *eba* = 4, *em* = 4 may suggest that *Euscorpius avcii* sp. n. is a species belonging to the *“tergestinus* complex”, but these are the only similar characters, in fact the morphology and the chaetotaxy reveal the great differences between these two species. *Euscorpius tergestinus* is larger in size (30-40 mm), it has a slender habitus with elongated pedipalps and DPS strongly developed, among the largest in the entire genus *Euscorpius*.

Its telson is very swollen, above average in both male and female. The metasomal carinae are much more pronounced, granulated and *Euscorpius avcii* sp. n. has a less swollen telson and the metasomal carinae almost smooth. The pedipalpal chela of *Euscorpius tergestinus* is slender and long, especially the fingers. In this species, trichobothrium *db* on the fixed finger is much more distal than in *Euscorpius avcii* sp. n. that has it in proximal position.

*Euscorpius tergestinus* has a more granulated carapace, and body, and developed furrows on the carapace whereas *Euscorpius avcii* sp. n. has almost smooth carapace, and body, with weak furrows, causing the appearance of a fairly flat carapace. The trichobothrial pattern of the pedipalp patella of *Euscorpius tergestinus* is reported as *Pv* = 7/11 (9), *Pe-et* = 5/8 (6 +) in Fet and Soleglad (2002). Based on this data, *Euscorpius avcii* sp. n. would fall within its range, but it actually does not. Fet and Soleglad (2002) synonymized *Euscorpius carpathicus oglasae*, with its low trichobothrial values, with *Euscorpius tergestinus*, but Vignoli et al. (2007) raised *Euscorpius oglasae* to the rank of species, therefore the range of *Euscorpius tergestinus* is *Pv* = 8/11 (9), *Pe-et* = 6/8 (6 +) (Tropea 2012). In fact, this species presents lowest values (*Pv* = 8, *Pe-et* = 6) in populations in central Italy (*Euscorpius carpathicus picenus*, *Euscorpius carpathicus apuanus*, of Di Caporiacco (1950)), however normally its trichobothrial numbers are *Pv* = 9 and *Pe-et* = 6. These values increase toward the northeast of Italy and in the Balkans (Tropea 2012), but they are never less, therefore *Euscorpius avcii* sp. n. does not share the trichobothrial range of *Euscorpius tergestinus* s. str.

Other species and subspecies of subgenus *Euscorpius* s.str. that are relatively geographically close, from the Aegean area: *Euscorpius sicanus* (C. L. Koch, 1837), *Euscorpius koshewnikowi* Birula, 1900, *Euscorpius carpathicus candiota* Birula, 1903, *Euscorpius carpathicus ossae* Di Caporiacco, 1950, *Euscorpius carpathicus aegaeus* Di Caporiacco, 1950 and *Euscorpius carpathicus scaber* Birula, 1900. *Euscorpius sicanus* has never been reported in Turkey; furthermore, it is easy to separate because of its particular trichobothrial pattern; *Pe*: *eb*=5 and *eba* = 4/5 (Fet et al. 2003; Vignoli and Salomone 2008; Tropea 2012). *Euscorpius koschewnikowi* has been well redescribed by Fet and Soleglad (2002) as a species quite large in size and medium to dark brown colored, exceptionally smooth, with all segments of the metasoma longer than wide, and DPS highly developed. The description of this species contrasts completely with *Euscorpius avcii* sp. n. because the latter is a small species, colored clear reddish brown, squat, with DPS very weakly developed, and not all metasomal segments are longer than wide. *Euscorpius carpathicus candiota*, among other differing characters, has a higher trichobothrial pattern as well as *Euscorpius carpathicus aegaeus* (Fet 1985; Di Caporiacco 1950), whereas *Euscorpius carpathicus ossae* is overall blackish with legs and telson slightly lighter and larger size (up to 37 mm) (Di Caporiacco 1950).

*Euscorpius carpathicus scaber* is a scorpion from the northern Aegean area, has a dark coloration with a high number of pectinal teeth, a higher trichobothrial pattern, and in addition, its whole body is covered with granules of various size, as also the name suggests, whereas *Euscorpius avcii* sp. n. has a light coloration, and its granulation is very little accentuated, almost smooth.

In our opinion, *Euscorpius avcii* sp. n. is well divided from all described *Euscorpius* forms including those that await taxonomic clarification. At present there are no described species or subspecies that corresponds to the morphology and to the trichobothrial pattern of this new species. We are confident that these data are enough to describe this form as a new species of the genus *Euscorpius*, and the first described species of the subgenus *Euscorpius* in Turkey.

**Table 1.** Measurements (in mm) of male holotype and female and male paratype of *Euscorpius avcii* sp. n.

** **		**Holotype**	**Paratype female**	**Paratype male**
**Total**	Length	26.18	23.65	27.70
**Carapace**	Length	3.70	3.60	3.90
	Posterior width	3.75	3.70	3.80
**Metasoma**	Length	9.78	8.20	10.10
**Segment I**	Length	1.25	1.10	1.30
	Width	1.40	1.30	1.40
**Segment II**	Length	1.50	1.35	1.60
	Width	1.20	1.10	1.20
**Segment III**	Length	1.75	1.45	1.80
	Width	1.15	1.05	1.10
**Segment IV**	Length	2.05	1.70	2.20
	Width	1.07	1.00	1.05
**Segment V**	Length	3.23	2.60	3.20
	Width	1.10	1.00	1.10
**Telson**	Length	3.65	2.85	3.70
**Vesicle**	Length	2.65	2.10	2.80
	Width	1.40	0.75	1.40
** **	Height	1.37	0.95	1.40
**Aculeus**	Length	1.00	0.75	0.90
**Femur**	Length	3.20	3.10	3.10
** **	Width	1.25	1.20	1.20
**Patella**	Length	3.25	3.20	3.45
** **	Width	1.20	1.25	1.30
**Chela**	Length	6.70	6.35	7.05
** **	Width	3.00	2.70	3.05
**Movable finger**	Length	3.85	3.10	4.00
**Pectines teeth**	** **	8–9	7–7	8–8

**Table 2.** Trichobothrial counts of *Euscorpius* species discussed in this paper.

**Species**	**v*P***	**t*Pe - e***	**t*Pe - es***	**m*Pe - e***	**b*Pe - es***	**a*Pe - eb***	**b*Pe - e***
*Euscorpius avcii* sp. n.	7	5–6	4	4	2	4	4
*Euscorpius oglasae*	7	5	4	4	2	4	4
*Euscorpius koschewnikowi*	8	5–6	4	4	2	4	4
*Euscorpius* sp. from*“Samos”*	5	5	4	4	2	4	4
*Euscorpius carpathicus aegaeus*	7–8(8)	5/6(6)	4	4	2	4	4
*Euscorpius carpathicus ossae*	6–8(7/8)	5	4	4	2	4	4
*Euscorpius carpathicus scaber*	7–10(8/9)	6	4	4	2	4	4
*Euscorpius carpathicus candiota*	9–10	6–7	4	4	2	4	4
*Euscorpius tergestinus*	8–11(9)	6–8(6)	4	4	2	4	4
*Euscorpius carpathicus* s.str.	7–9 (8)	5–7 (7)	4	3	2	4	4

## Ecology

**Figure 7.**
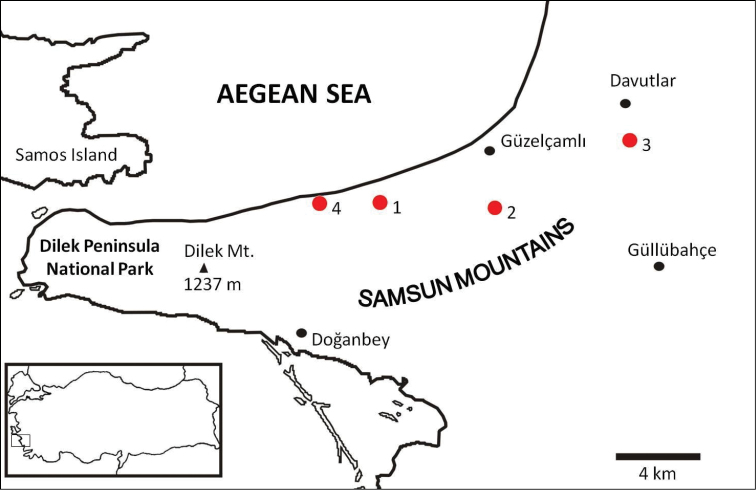
Map of Dilek Peninsula National Park

**Figure 8.**
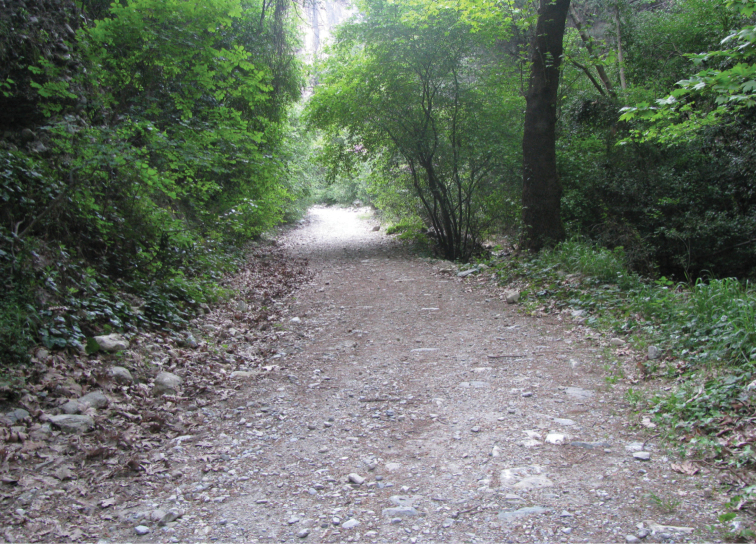
The habitat in Canyon in Dilek Peninsula National Park

**Figure 9.**
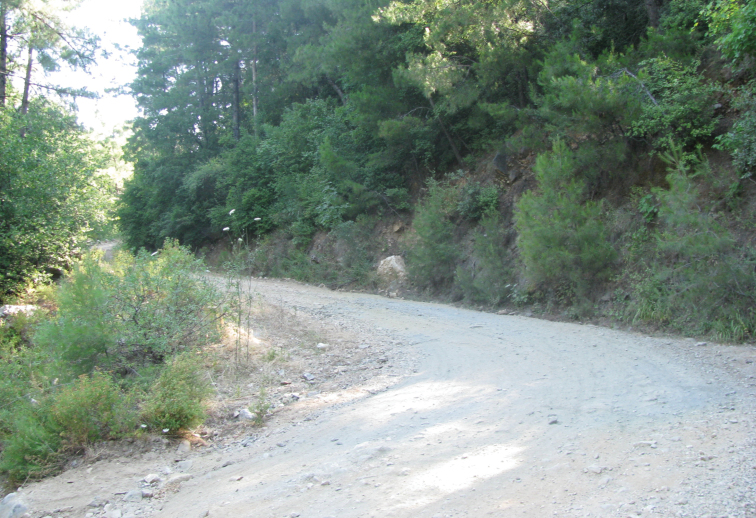
The habitat in North of Güzelçamlı in Dilek Peninsula.

**Figure 10.**
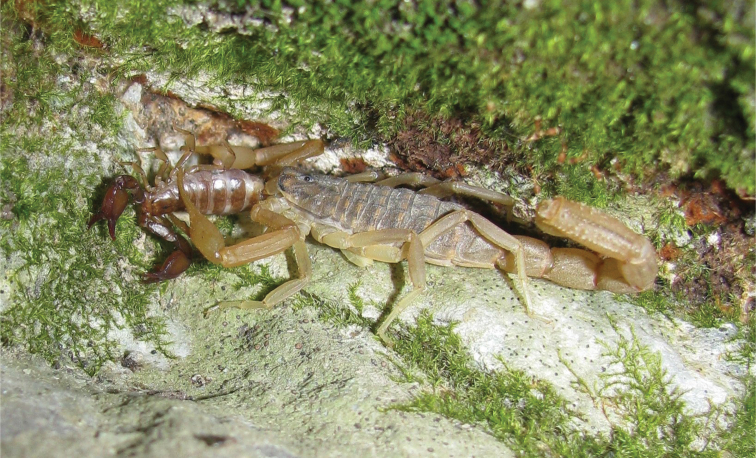
A *Mesobuthus gibbosus* which feeds on *Euscorpius avcii* sp. n.

Specimens of *Euscorpius avcii* sp. n. were collected from the northern side of Dilek Peninsula (Fig. 7). Vegetation in this area is composed of both deciduous forest (*Quercus cerris*, *Tilia rubra* subsp. *caucasica*, *Tilia argentea* and *Castanea sativa*) and evergreen forest (which are *Pinus brutia* and *Laurus nobilis*). Coastal areas include scrub vegetation. Furthermore, northern side of Dilek Peninsula has a humid climate and in both summer and winter, flowing streams and wetlands exist. Specimens of *Euscorpius avcii* sp. n. were collected during the day under bark of decomposed wood, under stones and in rock crevices and at night with UV light from rocky places, roadsides and under pine forests (Fig. 8 and 9). *Euscorpius avcii* sp. n. specimens are sympatric with *Mesobuthus gibbosus* Brullé, 1832 and *Iurus kinzelbachi* Kovarik, Fet, Soleglad, Yagmur, 2010. We report an example of intraguild predation, we witnessed *Mesobuthus gibbosus* feeding on *Euscorpius avcii* sp. n. during one of our night trips (Fig. 10).

## Supplementary Material

XML Treatment for Euscorpius avcii

## References

[B1] Di Caporiacco L (1950) Le specie e sottospecie del genere “*Euscorpius”* viventi in Italia ed in alcune zone confinanti. Memorie/Atti della Accademia Nazionale dei Lincei, serie VIII, volume II, sezione III, fascicolo 4: 159-230.

[B2] Fet V (1985) Notes on some *Euscorpius* (Scorpiones: Chactidae) from Greece and Turkey. Rivista del Museo Civico di Scienze Naturali “Enrico Caffi” (Bergamo) 9: 3-11.

[B3] Fet V (1997b): Notes on the taxonomy of some Old World scorpions (Scorpiones: Buthidae, Chactidae, Ischnuridae, (Scorpionidae). The Journal of Arachnology 25 (3): 245-250.

[B4] Fet V Braunwalder ME (2000) The scorpions (Arachnida: Scorpiones) of the Aegean area: current problems in taxonomy and biogeography. Belgian Journal Of Zoology 130(supplement 1): 15–20.

[B5] Fet V Soleglad ME (2002) Morphology analysis supports presence of more than one species in the ‘*Euscorpius carpathicus*’ complex (Scorpiones: Euscorpiidae). Euscorpius 3: 1-51.

[B6] Fet V Soleglad ME Gantenbein B (2004) The Euroscorpion: taxonomy and systematics of the genus *Euscorpius* Thorell, 1876 (Scorpiones: Euscorpiidae). Euscorpius 17: 47-60.

[B7] Fet V Soleglad ME Gantenbein B Vignoli V Salomone N Fet EV Schembri PJ (2003) New molecular and morphological data on the *Euscorpius carpathicus* species complex (Scorpiones: Euscorpiidae) from Italy, Malta, and Greece justify the elevation of *E. c. sicanus* (C.L. Koch, 1837) to the species level. Revues Suisse the Zoologie 110 (2): 355-379.

[B8] Gantenbein B Fet V Largiadèr CR Scholl A (1999) First DNA phylogeny of *Euscorpius* Thorell, 1876 (Scorpiones: Euscorpiidae) and its bearing on taxonomy and biogeography of this genus. Biogeographica (Paris) 75 (2): 49-65.

[B9] Hadži J (1930) Die europäischen Skorpione des Polnischen Zoologischen Staatsmuseums in Warszawa. Annales Musei Zoologici Polonici 9 (4): 29-38.

[B10] Hjelle JT (1990) Anatomy and morphology. In: Polis GA (Ed). Biology of Scorpions. Stanford University Press, Stanford, CA, 1990: 9-63.

[B11] Karatas A (2006) Distribution of the “*Euscorpius carpathicus*” complex (Scorpiones: Euscorpiidae) in Turkey. Serket 10 (1): 1-8.

[B12] Kinzelbach VR (1975) Die Skorpione der Ägäis. Beiträge zur Systematik, Phylogenie und Biogeographie. Zoologische Jahrbücher, Abteilung für Systematik 102: 12-50.

[B13] Kinzelbach R (1982) Die Skorpionssammlung des Naturhistorischen Museums der Stadt Mainz. Teil I: Europa und Anatolien. Mainzer Naturwissenschaftliches Archiv 20: 49-66.

[B14] Koç H Yağmur EA (2007) Dilek Yarımadasi Milli Parkı (Söke-Kuşadası, Aydın) akrep faunası. Ekoloji Dergisi 65: 52-59.

[B15] Koch CL (1837a) Die Arachniden. Nürnberg: C. H. Zeh‘sche Buchhandlung 3 (6): 105-115.

[B16] Lacroix J-B (1995) *Euscorpius* (*E*.) *mingrelicus* Kessler, 1876 en Turquie anatolienne (Arachnida: Scorpionida). Arachnides 26: 4-6.

[B17] Laurie M (1896) Further notes on the anatomy of some scorpions, and its bearing on the classification of the order. Annals and Magazine of Natural History 18 (6): 121-133. doi: 10.1080/00222939608680422

[B18] Schenkel E (1947) Einige Mitteilungen über Spinnentiere. Revue suisse de zoologie 54 (1): 13-16.

[B19] Sissom WD (1990) Systematics, biogeography and paleontology. In: Polis GA (Ed). The Biology of Scorpions. Stanford University Press, 1990: 64-160.

[B20] Soleglad ME Fet V (2003) The scorpion sternum: structure and phylogeny (Scorpiones: Orthosterni). Euscorpius 5: 1-33.

[B21] Soleglad ME Sissom WD (2001) Phylogeny of the family Euscorpiidae Laurie, 1896: a major revision. In: Fet V Selden PA (Eds). Scorpions. British Arachnological Society, Burnham Beeches, Bucks: 25-112.

[B22] Stahnke HL (1970) Scorpion nomenclature and mensuration. Entomological News 81: 297-316. 5417256

[B23] Thorell T (1876) On the classification of scorpions. Annals and Magazine of Natural History 4 (17): 1-15. doi: 10.1080/00222937608681889

[B24] Tolunay A (1959) Zur Verbreitung der Skorpione in der Türkei. Zeitschrift für angewandte Entomologie 43: 366-370.

[B25] Tropea G (2012) A new species of *Euscorpius* Thorell, 1876(Scorpiones, Euscorpiidae) from Italy. Bulletin of the British Arachnological Society 15 (8): 253-259

[B26] Vachon M (1951) A propres de quelques scorpions de Turquie collectés par M. le Professeur Dr. Curt Kosswig. İstanbul Üniversitesi Fen Fakültesi Mecmuası 16: 341-344.

[B27] Vachon M (1974) Étude des caractères utilisés pour classer les familles et les genres de Scorpions (Arachnides). 1. La trichobothriotaxie en Arachnologie, Sigles trichobothriaux et types de trichobothriotaxie chez les Scorpions. Bulletin du Muséum National d'Histoire Naturelle (Paris) 140: 857-958.

[B28] Vignoli V Salomone N (2008) A review of and additions to the current knowledge of the scorpion genus Euscorpius Thorell, 1876 (Scorpiones, Euscorpiidae). Fragmenta entomological 40 (2): 189-228.

[B29] Vignoli V Salomone N Cicconardi F Bernini F (2007) The scorpion of Montecristo, *Euscorpius oglasae* Di Caporiacco, 950, stat. nov. (Scorpiones, Euscorpiidae): a paleo-endemism of the Tuscan Archipelago (northern Tyrrhenian, Italy). Comptes Rendus Biologies 330: 113-125. doi: 10.1016/j.crvi.2006.11.003 17303538

